# Toward high-density streptavidin arrays on DNA origami nanostructures†

**DOI:** 10.1039/d5ra03393d

**Published:** 2025-07-14

**Authors:** Lukas Rabbe, Emilia Tomm, Guido Grundmeier, Adrian Keller

**Affiliations:** a Paderborn University, Technical and Macromolecular Chemistry Warburger Str. 100 33098 Paderborn Germany adrian.keller@uni-paderborn.de

## Abstract

The binding of the protein streptavidin (SAv) to biotin-modified DNA origami nanostructures (DONs) is widely employed in the single-molecule study of chemical reactions, the arrangement of functional proteins and nanomaterials with molecular precision, and numerous instances of DON-based cryptography, steganography, and computing. The latter application areas in particular would benefit from high-density SAv arrays to achieve higher information densities. The finite size and tetrameric nature of SAv, however, pose certain limits to the SAv density that can be achieved in such arrays. In this work, we explore these limits by investigating the impact of selected design factors and environmental conditions on SAv binding to DON-supported biotin arrays. We identify the optimal distance between neighboring binding sites and the optimal length of the single-stranded spacer between DON surface and biotin modification. This allows us to assemble a 2D SAv array composed of 20 biotin modifications arranged at a density of about 0.008 nm^−2^ with an average SAv–Bt binding yield of about 70%. Since higher binding yields are achieved for equivalent 1D arrays, our results suggest that molecular crowding is a major factor that limits the maximum binding yield achievable in such 2D arrays.

## Abstract 2

Streptavidin binding to DNA origami-supported high-density biotin arrays is investigated for selected experimental parameters. While bidentate binding and steric hindrance can be minimized, molecular crowding limits the binding yields in 2D arrays.

## Graphical abstract

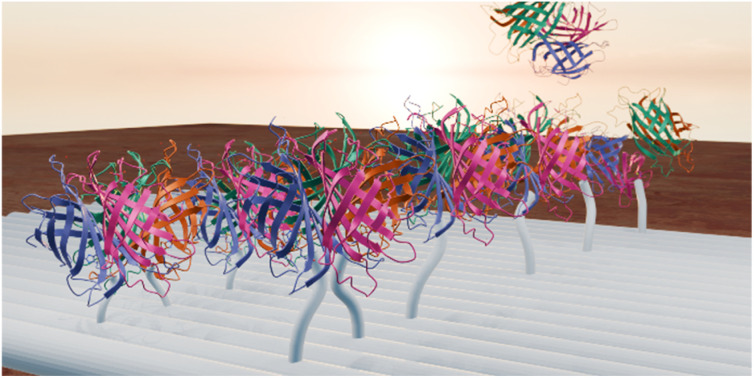

## 1. Introduction

In 2006, Rothemund introduced a technique for the folding of DNA into arbitrary yet well-defined nanostructures called DNA origami.^1^ Folding is achieved by the hybridization of a long single-stranded DNA scaffold with a large number of short synthetic staple strands. In addition to its unprecedented ability to assemble complex DNA nanostructures at near-stoichiometric yields, the DNA origami technique provides the unique possibility to arrange various functional molecules and nanoparticles with nanometer and sub-nanometer precision.^2^ This makes it an invaluable tool for a wide range of applications, ranging from drug delivery^3,4^ and drug discovery^2,5^ to sensing^6,7^ and plasmonics.^8,9^

Among the large variety of possible modifications of DNA origami nanostructures (DONs), the site-selective attachment of proteins has attracted particular attention. DONs have been decorated with numerous proteins,^10–23^ including various enzymes^12,15–17,22^ and antibodies.^13,18,19^ Protein decoration can be achieved using several different methods, including the covalent conjugation to DNA strands,^14,16,18,19,21,22^ the utilization of protein tags,^10,12,13,20^ and the binding to DNA-conjugated ligands.^10,11,15,17,21,23^ Among the latter, the specific binding of the protein streptavidin (SAv) to its ligand biotin (Bt) is a particularly popular strategy. One reason for this is the exceptionally strong binding of SAv to Bt. With a dissociation constant of 4 × 10^−14^ M, SAv–Bt binding is one of the strongest non-covalent interactions.^24^ Therefore, SAv is frequently used as a marker for visualizing the presence, modification, or intactness of staple overhangs on the DON surface by atomic force microscopy (AFM), for instance in cryptography and steganography,^25–27^ molecular computing,^28,29^ and single-molecule studies of chemical reactions.^30–32^ Furthermore, SAv is a homotetrameric protein consisting of four identical subunits (β barrels), each capable of binding one Bt molecule. It can thus be used as an adapter to attach biotinylated entities to biotinylated staple strands, enabling the display of functional proteins^11,15,17,21,23^ and nanomaterials^33–37^ on DONs.

In combination with its comparably large size of about 5 nm in diameter, this ability to participate in bidentate and multidentate binding events poses an intrinsic limitation for the density at which SAv can be displayed on DON surfaces. At high Bt densities, the distance between neighboring Bt modifications becomes so small, that a single SAv molecule can bind to several neighboring Bt sites.^5,38^ Furthermore, even in the absence of multidentate binding, the large size of SAv may result in reduced binding because of steric hindrance between neighboring proteins or between the proteins and the DON surface. These factors collectively may cause a pronounced reduction in the obtainable SAv–Bt binding yield at high Bt densities.

In this work, we therefore explore the limits of the SAv–Bt approach and investigate the impact of selected design factors and environmental conditions on SAv–Bt binding efficiency with the aim to achieve a high SAv density without compromising the binding yield. We identify the optimal distance between neighboring binding sites and the optimal length of the single-stranded spacer between DON surface and Bt modification. Using these parameters, we create a 4 × 5 SAv array with a binding site density of about 0.008 Bt modifications per nm^2^, corresponding to about 20% of a close packed square arrangement of SAv proteins, and an average SAv–Bt binding yield of about 70%.

## 2. Results and discussion

**Fig. 1:**
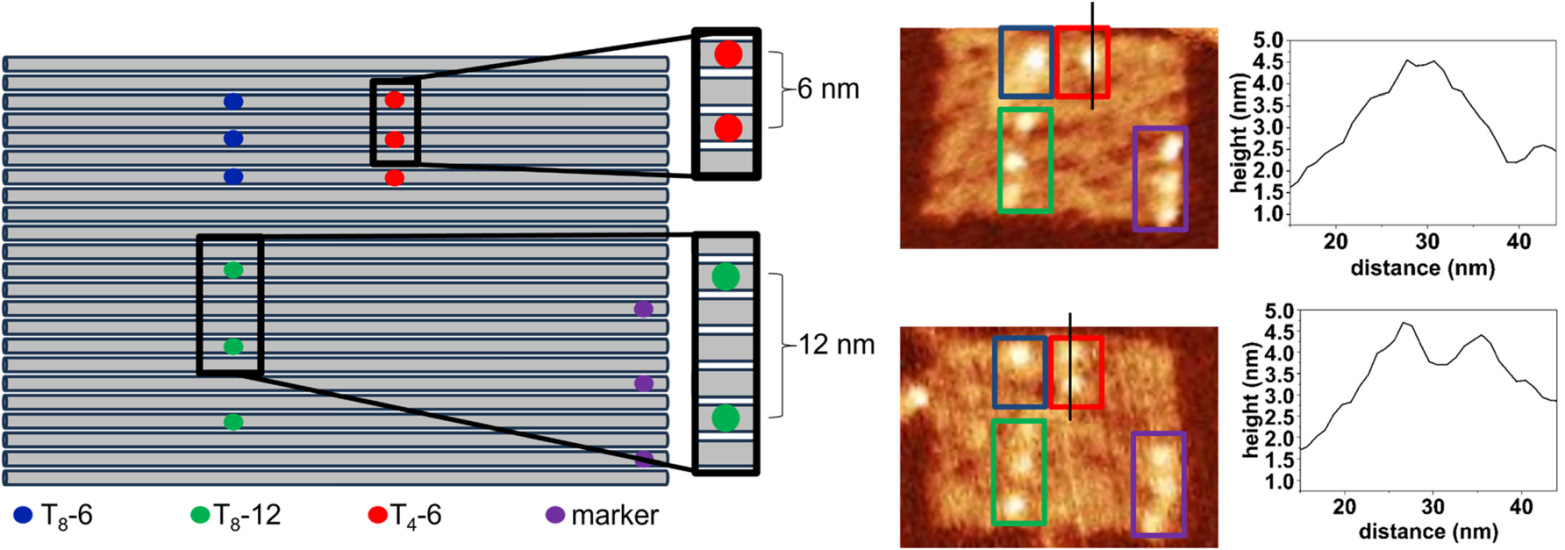
Arrangement of SAv binding sites on the DON rectangle (left) and example AFM images (right) with bound SAv molecules and highlighted binding sites. The height profiles were taken along the black lines indicated in the AFM images.

As the substrate for the high-density display of Bt modifications, we selected a twist-corrected DON rectangle with nominal dimensions of 65 × 95 nm.^39^ This rectangle features 168 nicks on one side (see Fig. S1 and S2†), rendering it an ideal substrate for this purpose. Three Bt arrangements were tested (Fig. 1). The closest distance between two neighboring Bt modifications is nominally 6 nm.^1^ SAv binding to three neighboring Bt modifications at this distance was investigated using two different single-stranded spacer sequences between the Bt modification and the hybridized staple consisting of four (T_4_-6, red) or eight thymines (T_8_-6, blue). The T_8_ spacer was also investigated at a larger binding site distance of nominally 12 nm (T_8_-12, green). To enable the unambiguous identification of the different binding sites, three additional bidentate binding sites were introduced, at which the 5′ and the 3′ ends of the two neighboring staples at each nick carry a T_4_ spacer with a Bt modification. Such bidentate binding sites have a higher affinity for SAv and are therefore used as markers to indicate the orientation and adsorption geometry of the DON.^5,38^

The folded, Bt-modified DONs were adsorbed on mica surfaces and subsequently exposed to SAv at varying concentrations for up to 30 min. After incubation, SAv binding was evaluated by AFM in the dry state after washing. In the example AFM images shown in Fig. 1, SAv molecules bound to the different binding sites can be clearly identified. This is further demonstrated by the height profiles taken across the T_4_-6 binding region, which in each case feature only two bound SAv molecules. In the lower AFM image, the two SAv molecules are bound to the two outer Bt sites, with the central one left empty. Therefore, two separated bumps can be resolved in both the image and the height profile. In the upper image, the two SAv molecules are bound to two neighboring sites. Despite imaging the sample with a pixel size of 1 nm^2^ and a tip with a 2 nm tip radius, the two neighboring proteins appear as a single, elongated bump. Nevertheless, the corresponding height profile reveals a broad peak with a full width at half maximum (fwhm) of about 12 nm, consistent with two SAv molecules bound to two Bt modifications with 6 nm distance. Therefore, in the quantitative analyses of SAv–Bt binding yields below, such height profiles were used to determine the number of bound proteins whenever visual inspection did not provide unambiguous results.

**Fig. 2:**
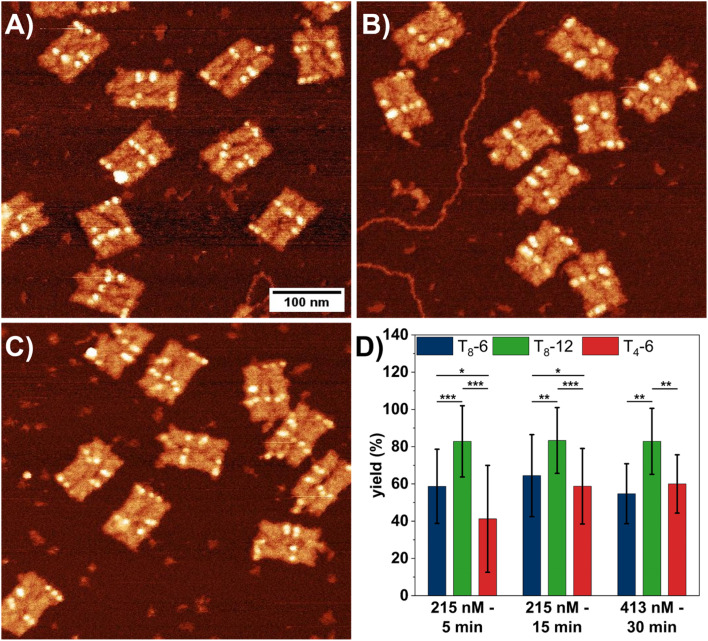
(A–C) AFM images (0.5 × 0.5 μm^2^) of DON rectangles after exposure to 215 nM SAv for 5 min (A), 215 nM SAv for 15 min (B), and 413 nM SAv for 30 min (C). (D) Corresponding SAv–Bt binding yields presented as mean values with the standard deviations as error bars. For each condition, 5 to 6 AFM images with a total of 70 to 105 DONs have been analyzed. Significances between the different Bt arrangements have been calculated using a two-sided *t* test with paired samples and are indicated as * (*p* < 0.05), ** (*p* < 0.01), and *** (*p* < 0.001).

First, we investigated the influence of SAv concentration and incubation time on the SAv–Bt binding yield for the three different Bt arrangements. Fig. 2A–C shows AFM images of DON rectangles after incubation with SAv at 215 nM for 5 (A) and 15 min (B), as well as at 413 nM for 30 min (C). The AFM images reveal that even at the highest SAv concentration of 413 nM, non-specific adsorption of SAv at the mica surface is negligible and does not impair image quality. SAv–Bt binding yields for each arrangement were determined by manual counting and are shown in Fig. 2D. For all three conditions, T_8_-12 shows the highest binding yield of about 83%, which does not show any dependence on SAv concentration and incubation time. The same is also observed for the shorter spacing, *i.e.*, T_8_-6, albeit at a lower value of about 60%. A notable dependence on the incubation conditions is observed only for the short spacer sequence, *i.e.*, T_4_-6. Here, the SAv–Bt binding yield increases from about 41% after 5 min incubation at 215 nM to about 60% after 15 min incubation at 215 nM. Increasing incubation time and SAv concentration to 30 min and 413 nM, respectively, does not yield any further improvements. Based on these observations, it appears that the incubation time has a larger effect on the binding yield than the SAv concentration, albeit only for the T_4_ spacer.

**Fig. 3:**
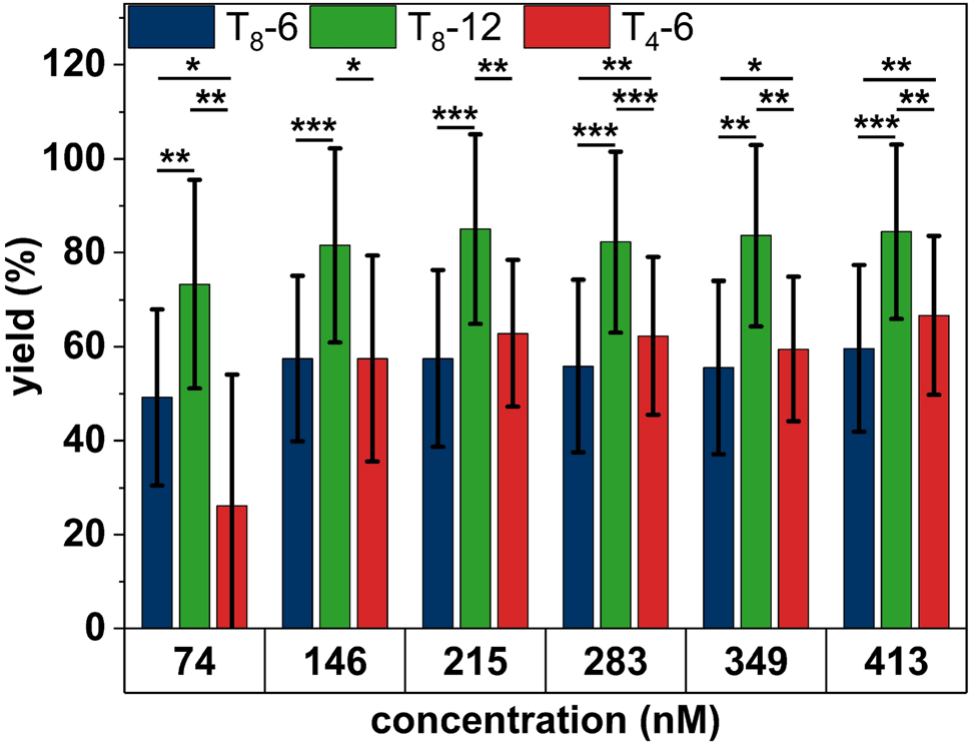
SAv–Bt binding yields for the stepwise addition of SAv presented as mean values with the standard deviations as error bars. For each condition, 4 to 10 AFM images with a total of 51 to 164 DONs have been analyzed. Significances between the different Bt arrangements have been calculated using a two-sided *t* test with paired samples and are indicated as * (*p* < 0.05), ** (*p* < 0.01), and *** (*p* < 0.001).

The data presented in Fig. 2 indicate that further increases in SAv concentration or incubation time will not result in higher binding yields, with a maximum yield around 80% achievable only for T_8_-12. Interestingly, similar binding yields around 80% are also observed for the bidentate marker positions (see Fig. S3†), which indicates that the sub-stoichiometric yields of the T_8_-12 sites are not caused by the spacer length. Therefore, we next tested whether an increase in SAv concentration during incubation leads to higher binding yields. Our approach is based on the published protocol by Woods *et al.*,^40^ in which the authors slowly ramped up the SAv concentration on the mica surface in an stepwise fashion to improve SAv–Bt binding. In our experiments, we added a volume of 1.5 μL of 5 μM SAv solution to 100 μL incubation buffer every 5 min for up to 30 min. This resulted in the stepwise increase of the SAv concentration depicted in Fig. S4.† After each 5 min incubation step, the samples were imaged and the SAv–Bt binding yields determined (Fig. 3).

Surprisingly, for T_8_-12, the obtained SAv–Bt binding yield is not much affected by the stepwise increase of the SAv concentration. After the first step at 74 nM SAv, a binding yield of about 73% is obtained. After the second step at 146 nM, this value increases slightly to about 80%, after which it remains constant. For the shorter distance, *i.e.*, T_8_-6, a similar yet less pronounced behavior is observed with an initial and saturated binding yield after the first and second step of about 50% and about 55%, respectively. As in the previous experiments, the shorter spacer, *i.e.*, T_4_-6, shows a stronger dependence on the experimental conditions, with its SAv–Bt binding yield increasing more dramatically from about 25% after the first step to about 60% after the third step. These observations not only demonstrate that the stepwise addition of SAv does not result in any improvement in SAv–Bt binding but also indicate that the arrangement of the Bt modifications has a stronger influence on the obtained binding yield than the environmental conditions.

**Fig. 4:**
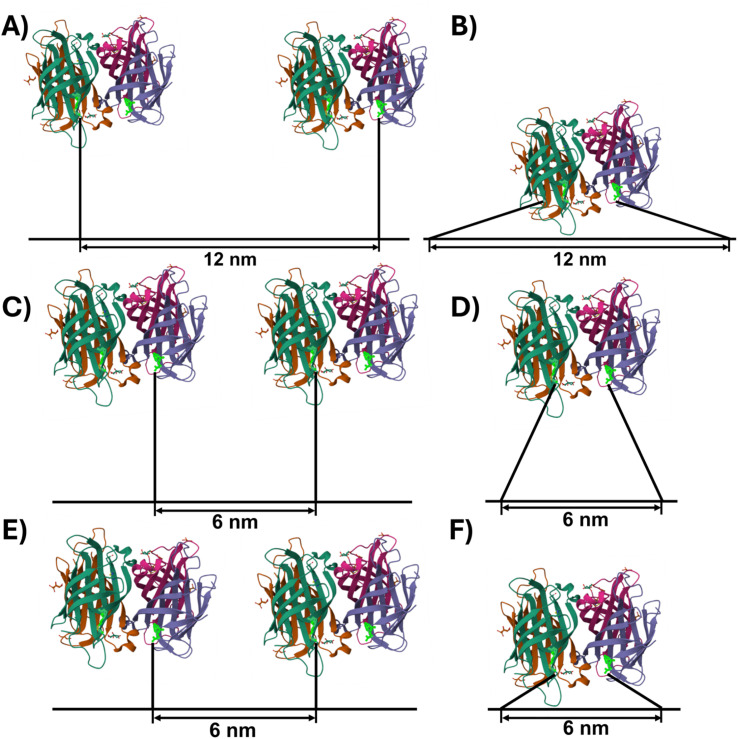
Schematic representations (to scale) of potential monodentate (A, C and E) and bidentate (B, D and F) SAv–Bt binding geometries. Spacer sequences are depicted fully stretched, resulting in a length of 4.8 nm and 2.4 nm for T_8_ (A–D) and T_4_ (E and F), respectively. The SAv structure with bound Bt (bright green) was taken from the RCSB PDB (https://www.rcsb.org), PDB ID 6J6J,^42^ and created using Mol* Viewer.^43^

To develop an understanding of the origins of the observed differences between the three Bt arrangements, Fig. 4 schematically depicts possible SAv–Bt binding geometries, assuming fully stretched T_8_ and T_4_ spacers with 0.6 nm per nucleotide. In the case of T_8_-12, monodentate binding of two SAv proteins to two neighboring Bt modifications is possible without any steric hindrance (Fig. 4A). In contrast, bidentate binding of one protein to two neighboring Bt modifications (Fig. 4B) does not seem possible without bending the DON surface. This, however, is highly unlikely after DON immobilization on the mica surface. Because of this and the fact that almost identical binding yields are observed also for the bidentate marker positions (see Fig. S3†), we rather assume that the obtainable binding yields are limited by intrinsic properties of the Bt-modified DON. Indeed, previous studies have shown that ligand immobilization on the DON surface can alter protein–ligand (including SAv–Bt) binding affinities.^2,41^ This was attributed to the influence of microenvironments on protein–ligand binding. Although the exact nature of this effect remains unknown, it was speculated that the DON's hydration shell and electric double layer play important roles in this regard, as those may overlap with the immobilized ligand and the hydration shell of the bound protein and thereby introduce additional repulsive interactions.^2^ This would also explain the sub-stoichiometric bidentate SAv binding to the marker positions (Fig. S3†).

At a shorter distance of 6 nm, steric hindrance between neighboring proteins will reduce the number of conformations that can be adopted in the monodentate binding geometry (Fig. 4C). At the same time, bidentate binding becomes more likely, as both the strain on the T_8_ spacers and steric hindrance is reduced (Fig. 4D). This will result in a reduced overall binding yield as observed in the experiments above. The shorter spacer length of T_4_-6 changes this situation slightly. Here, bidentate binding is again associated with strained spacers and some steric hindrance (Fig. 4F), whereas monodentate binding to two neighboring Bt modifications should still be possible without any additional steric hindrance between SAv and the DNA origami surface (Fig. 4E). Based on these simple geometric considerations, we would thus expect a slightly higher SAv–Bt binding yield for T_4_-6 than for T_8_-6. Indeed, this is observed in Fig. 3 for SAv concentrations ranging from 283 to 413 nM. The observation that at lower concentrations T_4_-6 exhibits lower binding yields than T_8_-6 may again be caused by properties of the microenvironment of the DON surface, such as the electric double layer that the SAv proteins need to cross in order to reach the Bt modifications.

**Fig. 5:**
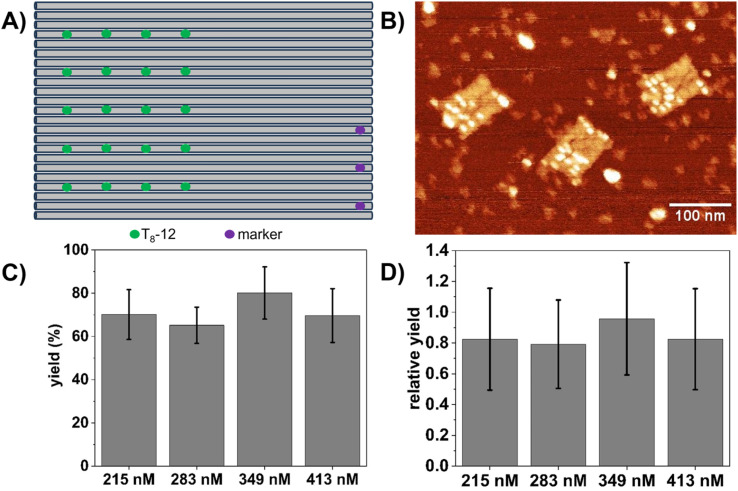
(A) Arrangement of 20 T_8_-12 SAv binding sites on the DON rectangle in a 2D array. (B) Corresponding AFM image after exposure to 413 nM SAv. (C) SAv–Bt binding yields for the stepwise addition of SAv presented as mean values with the standard deviation as error bars. For each condition, 5 to 6 AFM images with a total of 37 to 58 DONs have been analyzed. (D) Relative SAv–Bt binding yields obtained by normalizing the binding yields for 20 binding sites (panel C) to those for 3 binding sites (Fig. 3).

So far, only 1D columns of Bt sites arranged along the vertical DON axis in Fig. 1 were considered. Therefore, we next tested the Bt arrangement with the highest binding yield, *i.e.*, T_8_-12, in a 2D array consisting of 20 binding sites arranged in four parallel columns with a nominal distance between columns of about 11 nm (see Fig. 5A and B). These DONs were exposed to SAv by stepwise addition as in the previous experiments (Fig. 2) and SAv–Bt binding yields determined for final concentrations ranging from 215 to 413 nM (Fig. 5C). Within this range, no clear influence of SAv concentration on the binding yield is observed, with binding yields fluctuating around 70%. This is an additional reduction in binding yield by up to 20% compared to the linear arrays (see Fig. 5D). This indicates that even for large distances > 10 nm between binding sites, molecular crowding plays a role and may cause a notable reduction in binding yield, presumably *via* the restriction of lateral movement of bound and unbound proteins. First, within 1D arrays, steric hindrance between neighboring proteins may be compensated for by a lateral movement of the bound proteins in the direction perpendicular to the axis of the Bt modifications. In 2D arrays, this lateral movement is hindered by the presence of bound SAv molecules in the neighboring Bt columns. Second, the presence of bound proteins in the direct vicinity of an unoccupied binding site restricts its accessibility for incoming, unbound SAv molecules, as those can no longer diffuse laterally along the DON surface until they find an unoccupied Bt binding site but rather have to penetrate the array at the location of the Bt site. This situation is thus similar to diffusion of a protein into a nanopore that is only slightly larger than the protein itself.

## 3. Conclusion

In summary, we have studied the effect of several factors on the binding of SAv to Bt modifications arranged on the surface of a DON rectangle with the aim of achieving high-density SAv arrays. In general, our experiments revealed that design factors such as the distance between neighboring binding sites, the length of the single-stranded spacers, and the dimensionality of the array have a much stronger impact on the achievable binding yields than environmental conditions. Maximum SAv–Bt binding yields of about 80% are obtained for 1D arrangements of T_8_ spacers at a distance of 12 nm. Shorter spacers and shorter distances both lead to reduced binding yields. Most importantly, 2D arrays with identical spacers and distances were found to exhibit lower SAv–Bt binding yields of only about 70% due to molecular crowding effects.

Our results demonstrate that there is a limit to the density of SAv arrays that can be assembled at high yields on DON surfaces. Whereas the impact of bidentate binding events and steric hindrance can be minimized by appropriate combinations of binding site distances and spacer lengths, molecular crowding becomes a limiting factor in large 2D arrays. Additionally, the microenvironment on the DON surface may cause further reductions in the obtained binding yields. Using the optimal parameters identified in our experiments, we created a 4 × 5 SAv array with an average SAv–Bt binding yield of about 70%. This array has a binding site density of about 0.008 Bt molecules per nm^2^, corresponding to about 20% of a close packed square arrangement of SAv proteins (about 0.04 nm^−2^). Based on our experiments, we have to assume that attempts to increase the SAv density any further will have to face notably reduced binding yields due to steric hindrance and more bidentate binding events.

## 4. Materials and methods

### 4.1 DNA origami assembly and purification

The twist-corrected DON rectangles^39^ were folded at a ten-fold excess of staples (Eurofins) to scaffold (p7249, Tilibit). For SAv–Bt binding, selected non-modified staples were replaced with their biotinylated counterparts (HPLC-purified, Metabion). Folding was done in 1× TAE-buffer (Tris-acetate-EDTA, Carl Roth) with 10 mM MgCl_2_ (Carl Roth) in a thermocycler (VWR Ristretto or Bio-Rad T100) as described previously.^44^ The assembled DONs were purified by spin-filtering with Amicon Ultra-0.5 filters (100 kDa MWCO, Millipore) in a Mini-Spin centrifuge (Eppendorf) as described previously.^45^ The concentration of the folded and purified DONs were determined by UV/Vis absorption using an Implen Nanophotometer P330.

### 4.2 DON adsorption on mica surfaces and SAv exposure

The purified DONs were diluted to 1 nM concentration in 1× TAE containing 10 mM MgCl_2_ and immobilized on freshly cleaved mica surfaces by applying 100 μL of the DON solution. After 5 min of incubation, the surfaces were washed three times with 4 mL of 1× TAE with 10 mM MgCl_2_ and dried in a stream of argon. Afterwards, 100 μL of 1× TAE with 10 mM MgCl_2_ were deposited on the surfaces, followed by 5 μM SAv (Sigma Aldrich) dissolved in HPLC-grad water. The volume of SAv added was adjusted to yield the desired total concentration. For the stepwise addition of SAv, 1.5 μL aliquots were added in 5 min intervals. After the incubation for the desired time or number of steps, the surfaces were washed three times with 4 mL HPLC grade water and dried in a stream of argon.

### 4.3 AFM imaging and statistical analysis

AFM imaging was performed in air using a Dimension Icon (Bruker) operated in ScanAsyst mode with ScanAsyst-Air cantilevers (nominal tip radius 2 nm, Bruker). Images were recorded at a size of 1 × 1 μm^2^, a resolution of 1024 × 1024 px, and a line rate of 1 Hz. The images were processed with Gwyddion.^46^ SAv–Bt binding yields were determined by manually counting the bound proteins per binding site for each image. The obtained values were averaged over all AFM images recorded for each condition. Binding yields are given as mean values with the standard deviations as error bars. Statistical significance was evaluated by calculating *p* values in Excel (Microsoft 365) using a two-sided *t* test with paired samples.

## Author contributions

Lukas Rabbe: Formal analysis, investigation, methodology, validation, visualization, writing – original draft, writing – review and editing; Emilia Tomm: Investigation, methodology, writing – review and editing; Guido Grundmeier: Resources, writing – review and editing, supervision; Adrian Keller: Conceptualization, funding acquisition, methodology, writing – review and editing, supervision.

## Conflicts of interest

There are no conflicts to declare.

## Supplementary Material

RA-015-D5RA03393D-s001

## Data Availability

Data for this article, *i.e.*, raw AFM images, are available at Zenodo at https://doi.org/10.5281/zenodo.15370091.
