# Supplementary material for: Toward high-density streptavidin arrays on DNA origami nanostructures
Source: RSC Adv. 2025 Jul 14;15(30):24536–43. doi: 10.1039/d5ra03393d (PMC12257290; doi:10.1039/d5ra03393d)
Supplement: RA-015-D5RA03393D-s001 [file RA-015-D5RA03393D-s001.pdf]

Supplementary Information (SI) for RSC Advances.
This journal is © The Royal Society of Chemistry 2025

Supplementary Information

Toward high-density streptavidin arrays on DNA origami nanostructures

Lukas Rabbe, Emilia Tomm, Guido Grundmeier, and Adrian Keller

Table S1: Sequences of biotinylated staples used for testing different biotin arrangements. Spacer sequences are indicated in boldface.

Label	position [start]	position [end]	biotinylated staples
T_8-6	2[111]	0[112]	[biotin]**TTTTTTTT**GGGTGAGATTATTTCAACGCAAGGTAACCTGT
T_8-6	4[111]	2[112]	[biotin]**TTTTTTTT**GCATTAAACGGTTGATAATCAGAAATTCAAAA
T_8-6	6[111]	4[112]	[biotin]**TTTTTTTT**CTGGTGCCGTAACCGTGCATCTGCTAAAATTC
T_4-6	2[175]	0[176]	[biotin]**TTTT**TAGTCAGAGAAGCCCGAAAGACTTTCCCAA
T_4-6	4[175]	2[176]	[biotin]**TTTT**TCAACTAAGCATAGTAAGAGCAAACTATTA
T_4-6	6[175]	4[176]	[biotin]**TTTT**GACAAGAAAGCTGCTCATTCAGTACCACAT
T_8-12	12[111]	10[112]	[biotin]**TTTTTTTT**AATGGATTTAATATCCAGAACAATTCCACGCT
T_8-12	16[111]	14[112]	[biotin]**TTTTTTTT**CCAGAAGGCGACAACTCGTATTAACAAATGAA
T_8-12	20[111]	18[112]	[biotin]**TTTTTTTT**CTTAGGTTCGATAGCTTAGATTAAGCGAATTA
Marker	14[271]	12[272]	[biotin]**TTTT**AAGCGCAGTACCAGGCGGATAAGGGAATAG
Marker	16[271]	14[272]	CGTCAGACACGATTGGCCTTGATGAATGGA**TTTT**[biotin]
Marker	18[271]	16[272]	[biotin]**TTTT**GGAATAAGAACCATCGATAGCAGCCTTTAG
Marker	20[271]	18[272]	TGAGCGCTTAGAAAATACATACAACACCAC**TTTT**[biotin]
Marker	22[271]	20[272]	[biotin]**TTTT**GCGGGAGGAGCGCATTAGACGGGGGTAAT
Marker	23[256]	22[272]	AGATATAGAAGGCTTATCCGGTACCGACTT**TTTT**[biotin]

Table S2: Sequences of biotinylated staples used for generating the 4 x 5 biotin array. Spacer sequences are indicated in boldface.

Label	position [start]	position [end]	biotinylated staples
T_8-12	4[47]	2[48]	[biotin]**TTTTTTTT**GCGTCTGGTGGAGCAAACAAGAGATAGCTGAT
T_8-12	4[79]	2[80]	[biotin]**TTTTTTTT**TTAACCAACGTAAAACTAGCATGAAATCAC
T_8-12	4[111]	2[112]	[biotin]**TTTTTTTT**GCATTAAACGGTTGATAATCAGAAATTCAAAA
T_8-12	4[143]	3[159]	[biotin]**TTTTTTTT**AATTGTAAGAAGATTGTATAAGCACAAAAGGA
T_8-12	8[47]	6[48]	[biotin]**TTTTTTTT**TGAGTGAGGGTAACGCCAGGGTTTGGAAGGGC
T_8-12	8[79]	6[80]	[biotin]**TTTTTTTT**GAAGCATAGTAAAACGACGGCCAGCCATTC
T_8-12	8[111]	6[112]	[biotin]**TTTTTTTT**CCGCTCACCCTGCAGGTCGACTCTCACCGCTT
T_8-12	8[143]	7[159]	[biotin]**TTTTTTTT**GTCATAGCACCGAGCTCGAATTCGAGAGGCAA
T_8-12	12[47]	10[48]	[biotin]**TTTTTTTT**CCAACAGAACGTGGACTCCAACGTTCCCTTAT
T_8-12	12[79]	10[80]	[biotin]**TTTTTTTT**TCACACGAGTCTATCAACTCAAACTGTTTG
T_8-12	12[111]	10[112]	[biotin]**TTTTTTTT**AATGGATTTAATATCCAGAACAATTCCACGCT
T_8-12	12[143]	11[159]	[biotin]**TTTTTTTT**AATACCTATGCAACAGGAAAAACGTGTACCGT
T8-12	16[47]	14[48]	[biotin]**TTTTTTTT**TCCTGATTGATTAGAGCCGTCAATGGTCAGTT
T_8-12	16[79]	14[80]	[biotin]**TTTTTTTT**GATTATCAGATTTAGAAGTATTAACCTCAA
T_8-12	16[111]	14[112]	[biotin]**TTTTTTTT**CCAGAAGGCGACAACTCGTATTAACAAATGAA
T_8-12	16[143]	15[159]	[biotin]**TTTTTTTT**AACATTATGTTATTAATTTTAAAAAGAGCCGC
T_8-12	20[47]	18[48]	[biotin]**TTTTTTTT**GCGAGAAATAACCTTGCTTCTGTAAATTAATT
T_8-12	20[79]	18[80]	[biotin]**TTTTTTTT**GCTGATGCTAATTTTCCCTTAGAGAAGATG
T_8-12	20[111]	18[112]	[biotin]**TTTTTTTT**CTTAGGTTCGATAGCTTAGATTAAGCGAATTA
T_8-12	20[143]	19[159]	[biotin]**TTTTTTTT**AGGTCTGACAATAGTGAATTTATCGATAGCCG

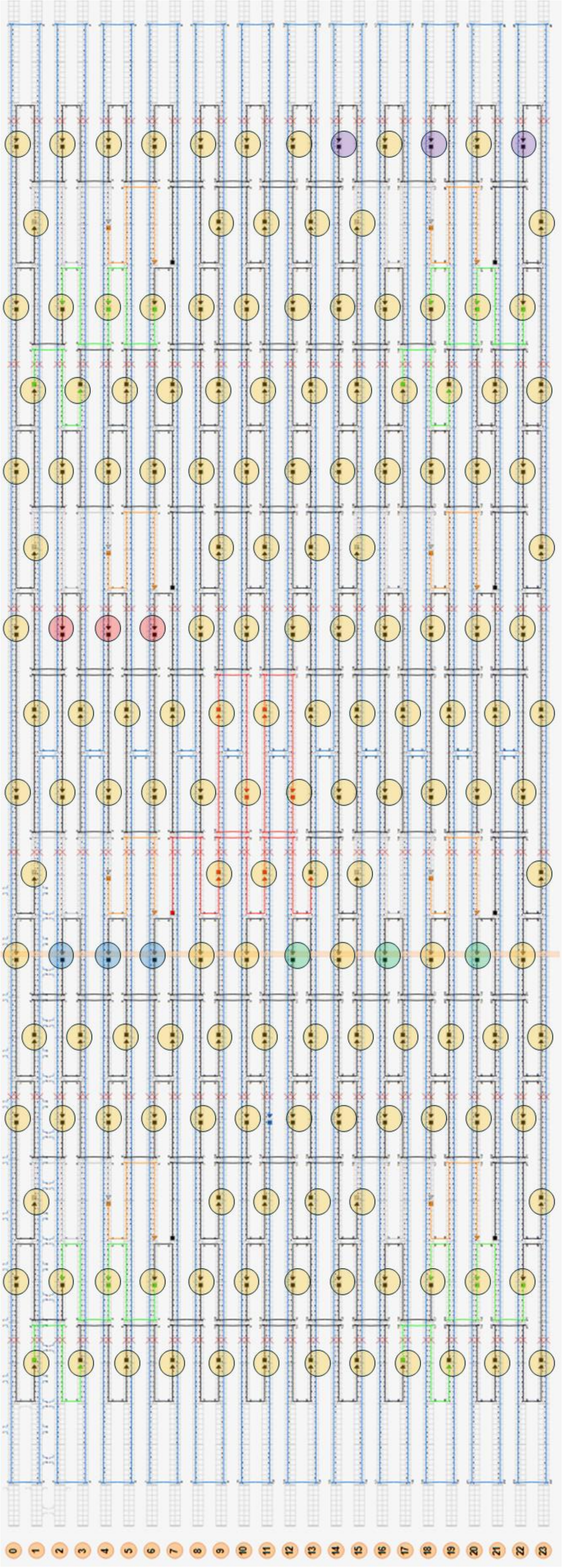

Figure S1: Schematic CaDNAno representation of the twist-corrected DON rectangle. All staple nicks located on the same side are highlighted. Those highlighted in red (T_4-6), blue (T_8-6), green (T_8-12), and purple (marker) were used for testing different Bt arrangements.

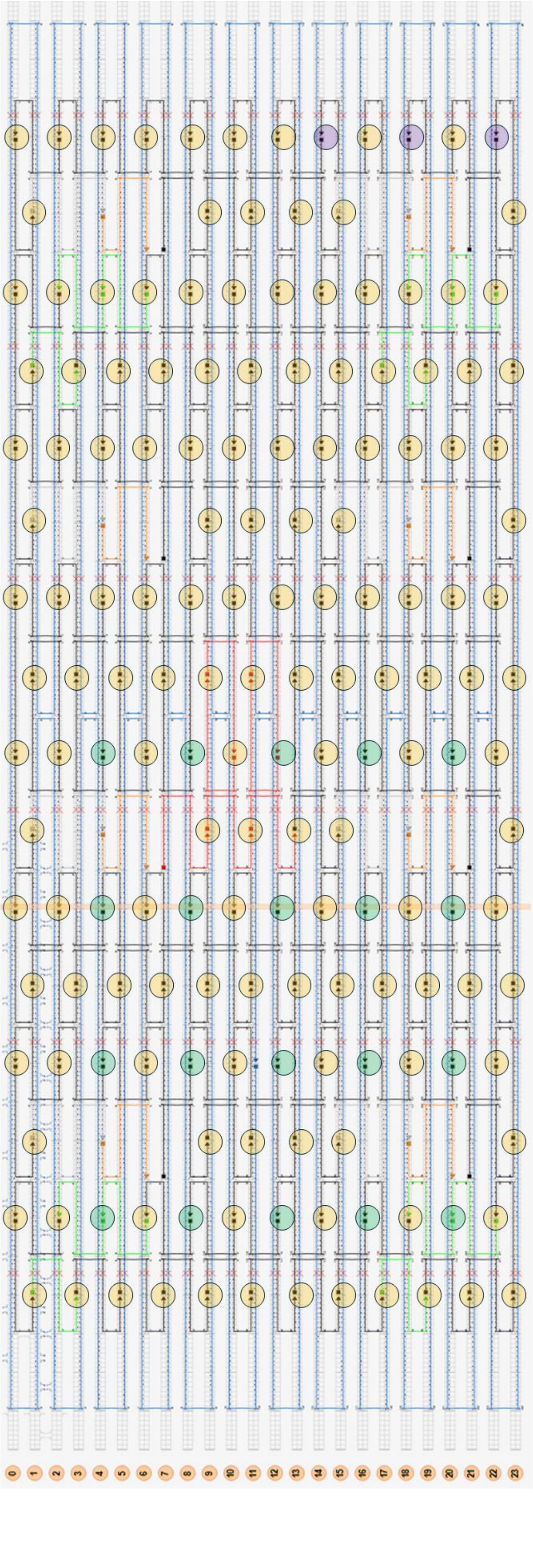

Figure S2: Schematic CaDNAno representation of the twist-corrected DON rectangle. All staple nicks located on the same side are highlighted. Those highlighted in green were used for generating the 4 x 5 Bt array.

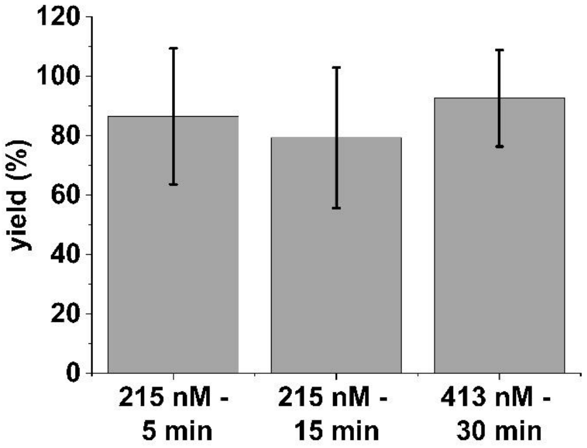

Figure S3: Bidentate SAv-Bt binding yields of the marker positions under the same conditions as shown in Figure 2. The data are presented as mean values with the standard deviations as error bars. For each condition, 6 AFM images with a total of 70 to 105 DONs have been analyzed.

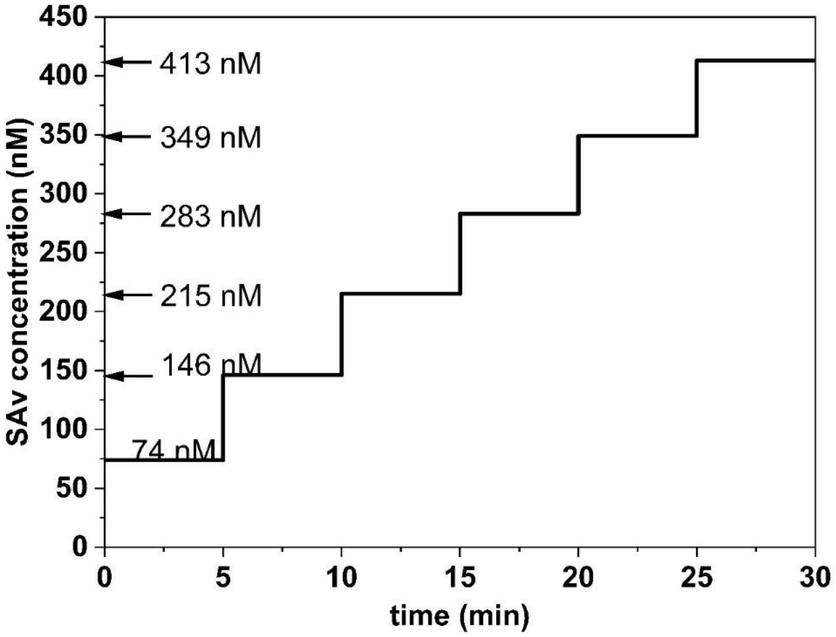

Figure S4: Increase of the SAv concentration over time due to stepwise addition of 1.5 µL of 5 µM streptavidin solution to 100 µL incubation buffer in 5 min increments.

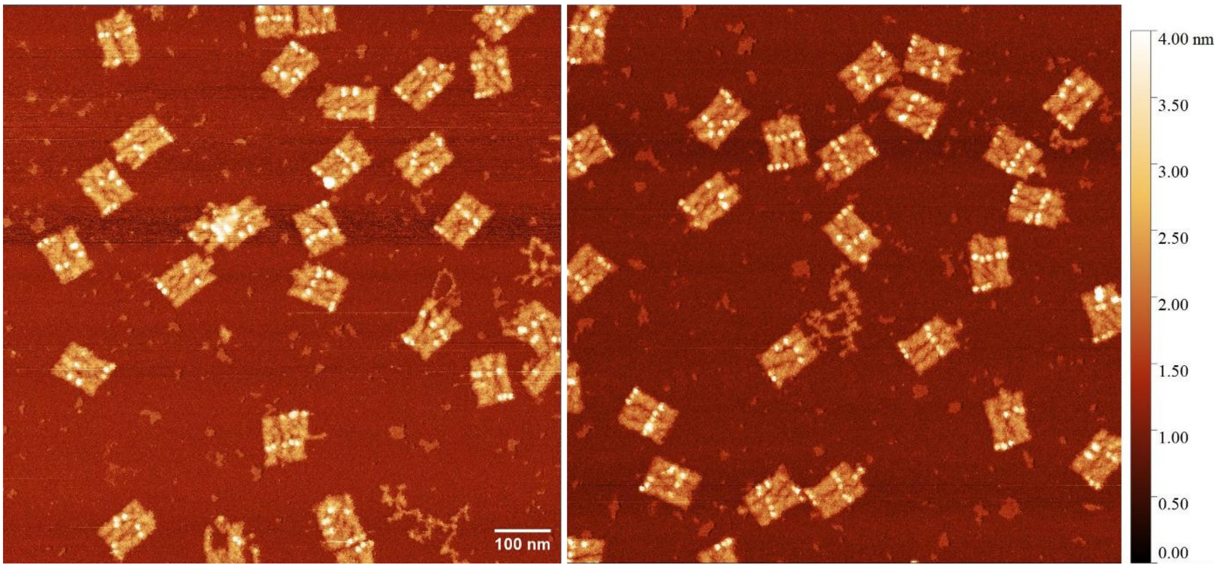

Figure S5: AFM images of DON rectangles with T_4-6, T_8-6, and T_8-12 Bt modifications after incubation with 215 nM SAv for 5 min.

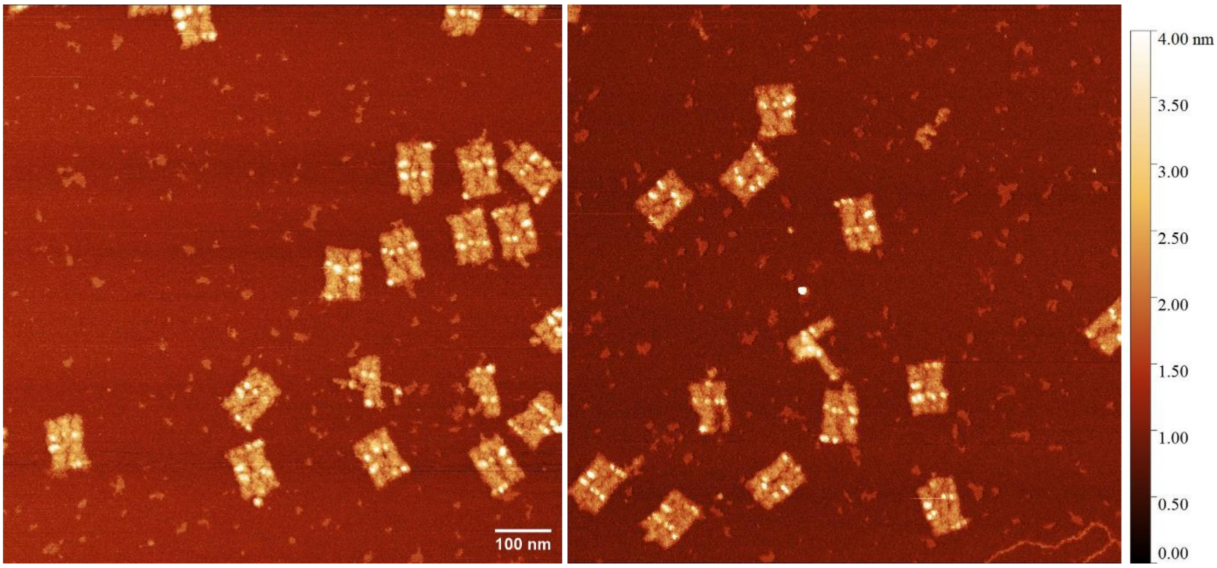

Figure S6: AFM images of DON rectangles with T_4-6, T_8-6, and T_8-12 Bt modifications after incubation with 215 nM SAv for 15 min.

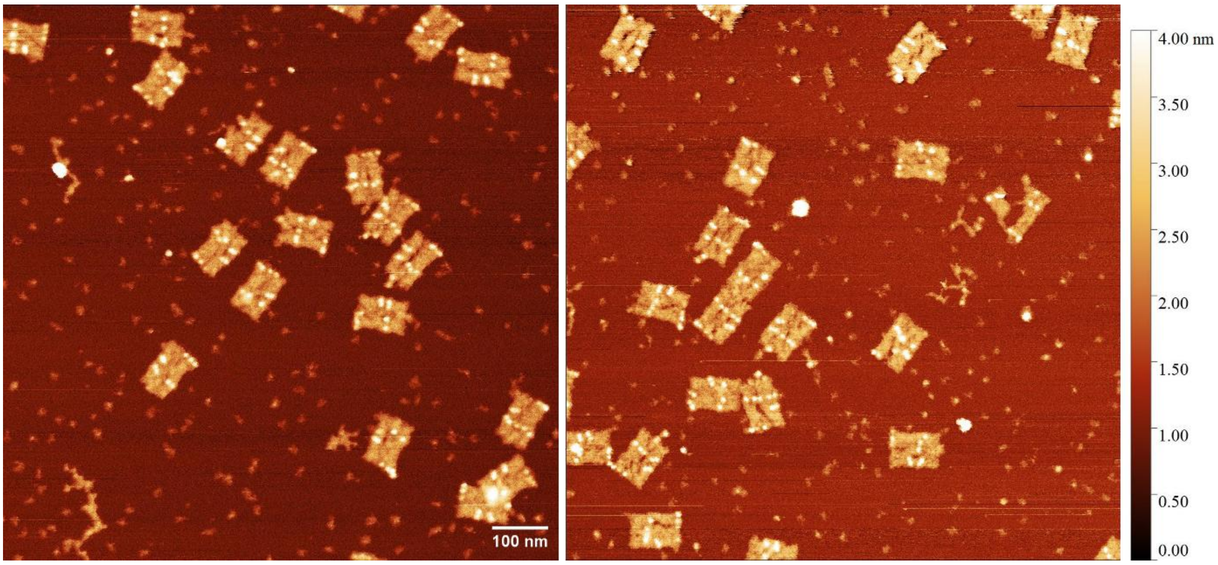

Figure S7: AFM images of DON rectangles with T_4-6, T_8-6, and T_8-12 Bt modifications after incubation with 413 nM SAv for 30 min.

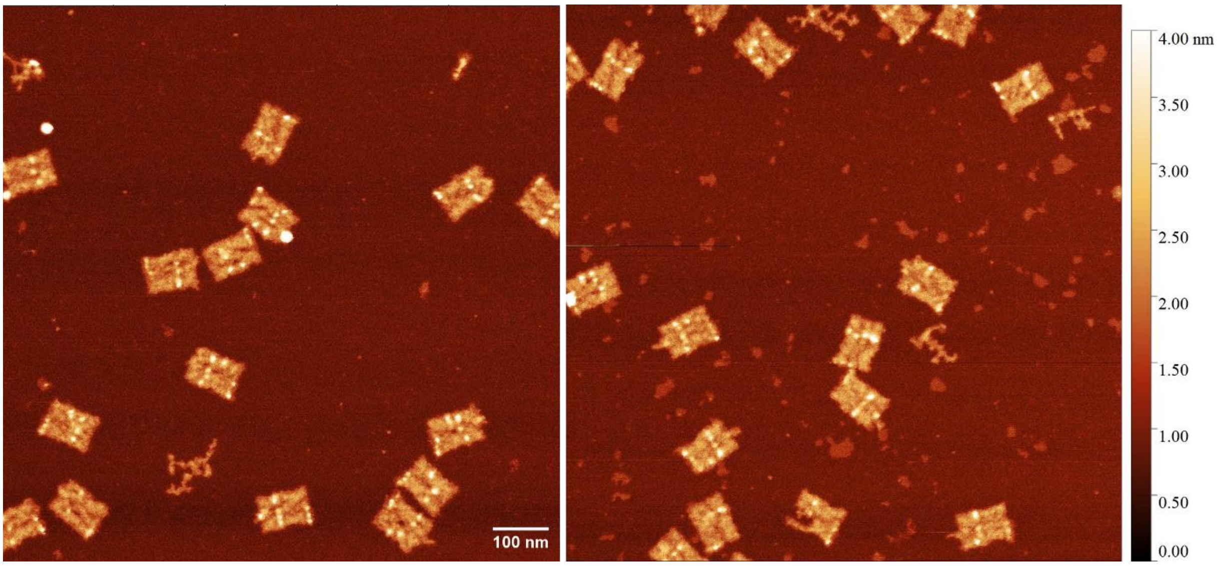

Figure S8: AFM images of DON rectangles with T_4-6, T_8-6, and T_8-12 Bt modifications after stepwise SAv addition at a final concentration of 74 nM.

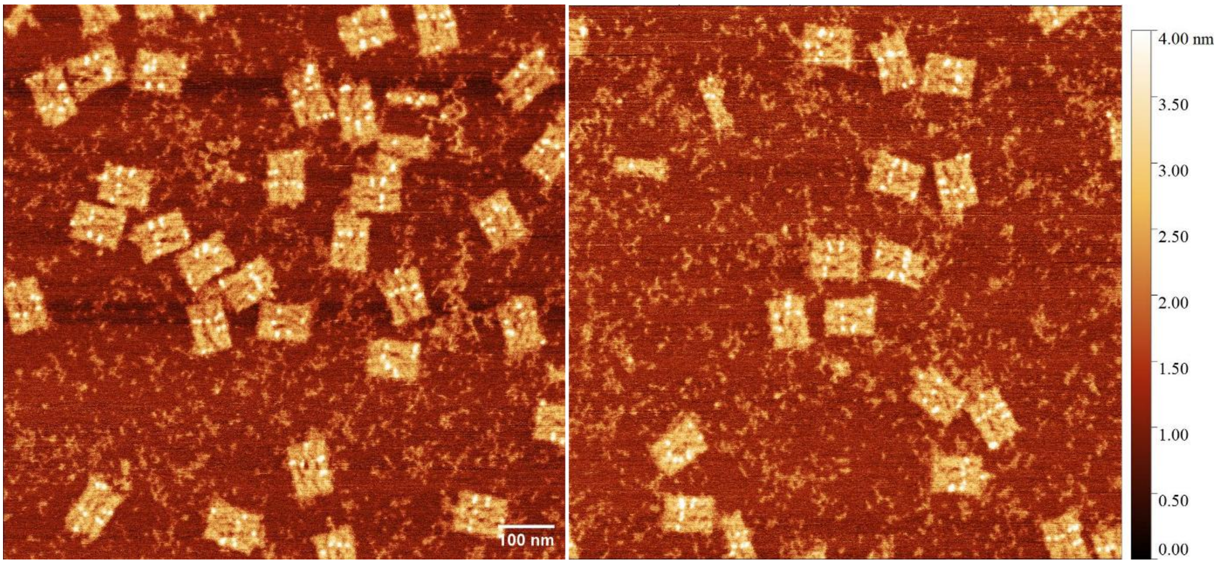

Figure S9: AFM images of DON rectangles with T_4-6, T_8-6, and T_8-12 Bt modifications after stepwise SAv addition at a final concentration of 146 nM.

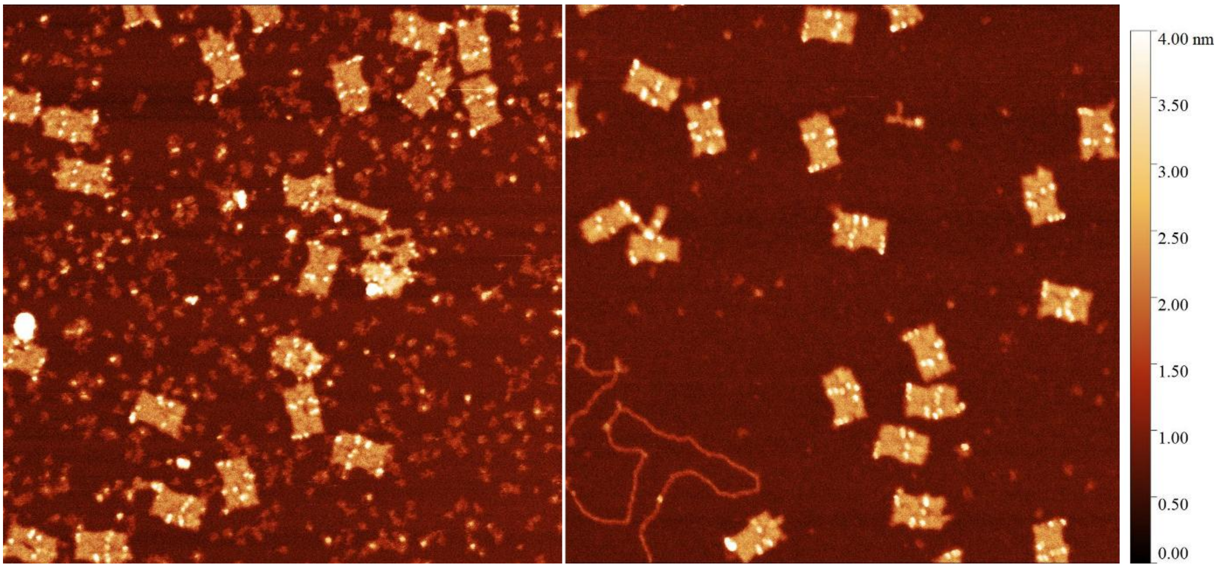

Figure S10: AFM images of DON rectangles with T_4-6, T_8-6, and T_8-12 Bt modifications after stepwise SAv addition at a final concentration of 215 nM.

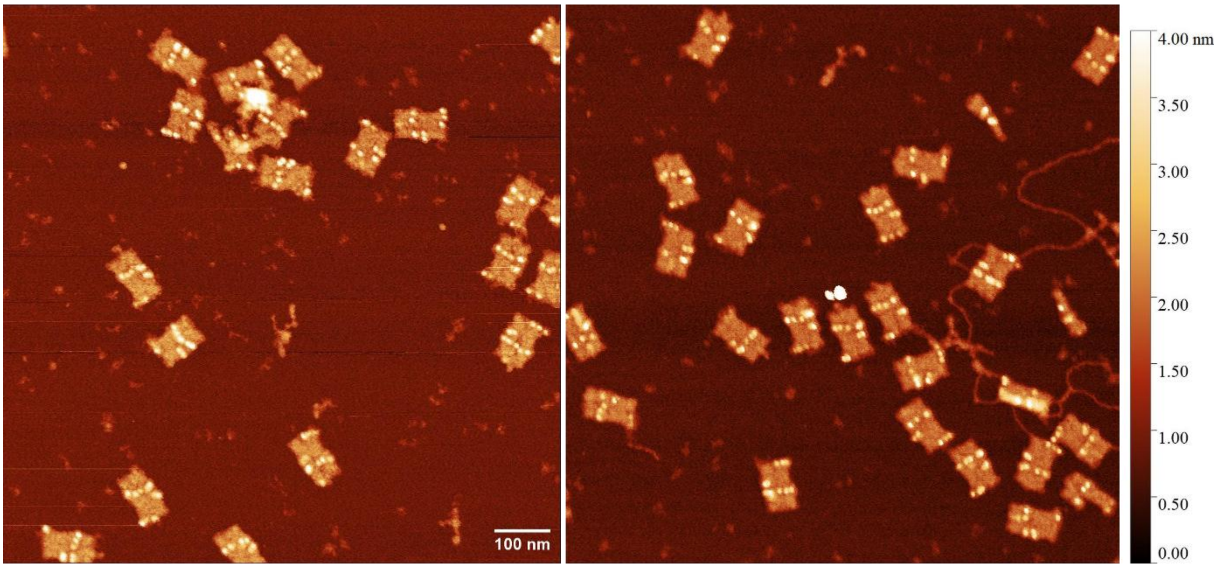

Figure S11: AFM images of DON rectangles with T_4-6, T_8-6, and T_8-12 Bt modifications after stepwise SAv addition at a final concentration of 283 nM.

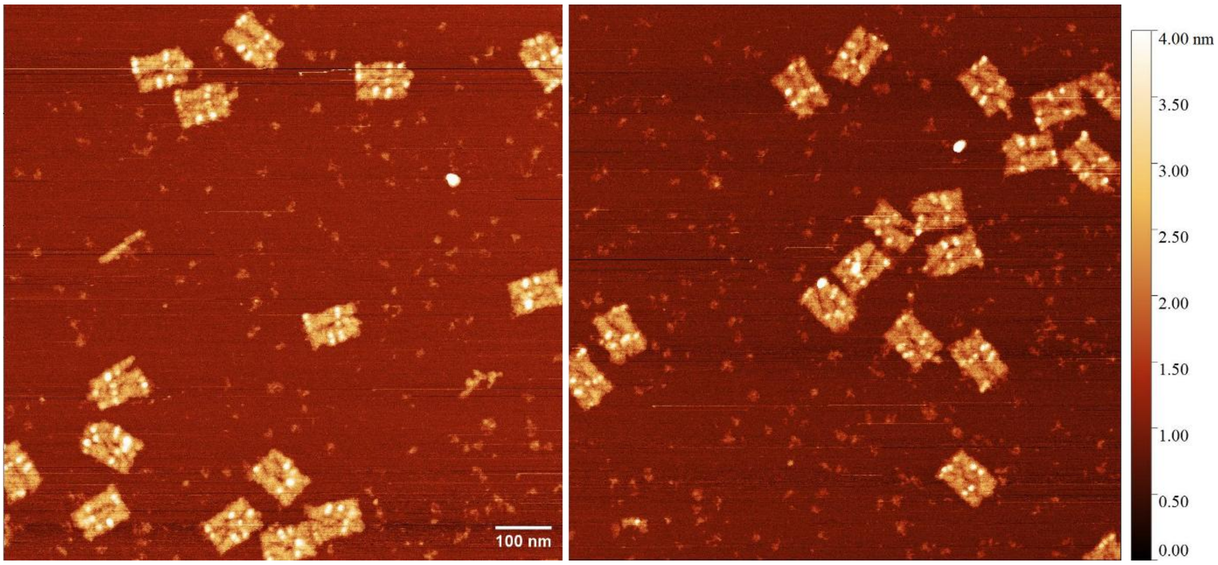

Figure S12: AFM images of DON rectangles with T_4-6, T_8-6, and T_8-12 Bt modifications after stepwise SAv addition at a final concentration of 349 nM.

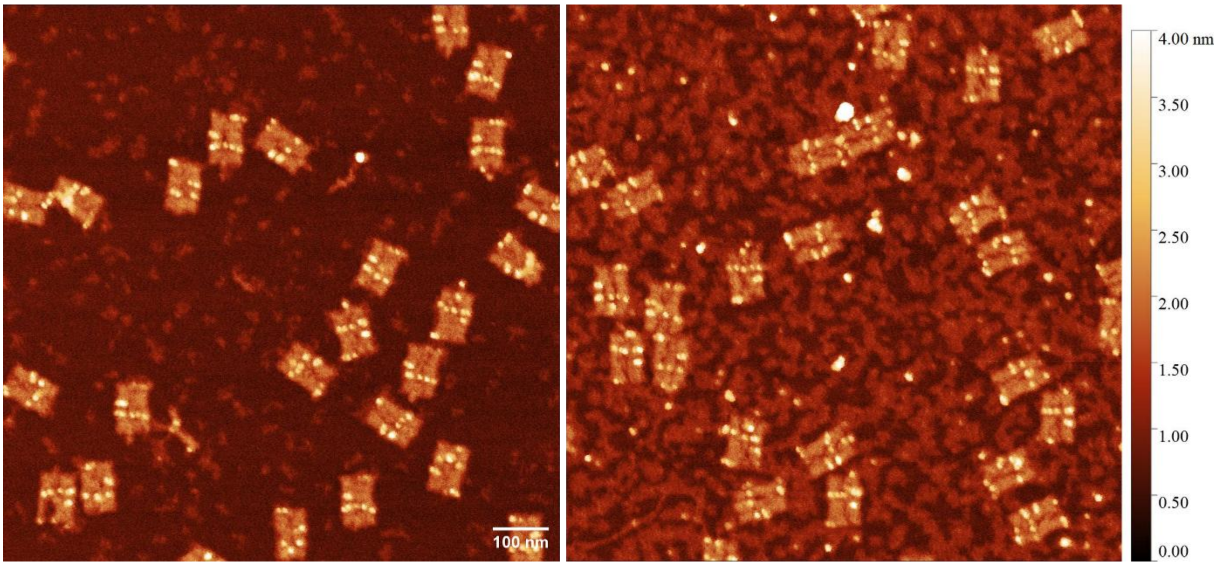

Figure S13: AFM images of DON rectangles with T_4-6, T_8-6, and T_8-12 Bt modifications after stepwise SAv addition at a final concentration of 413 nM.

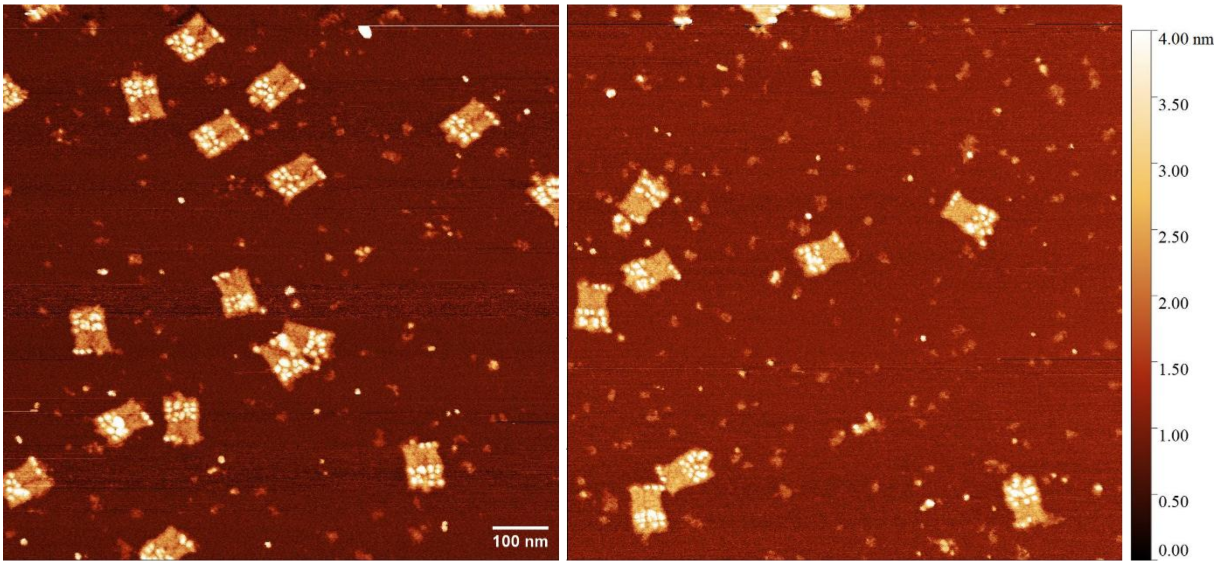

Figure S14: AFM images of DON rectangles with the 3 x 4 Bt array after stepwise SAv addition at a final concentration of 215 nM.

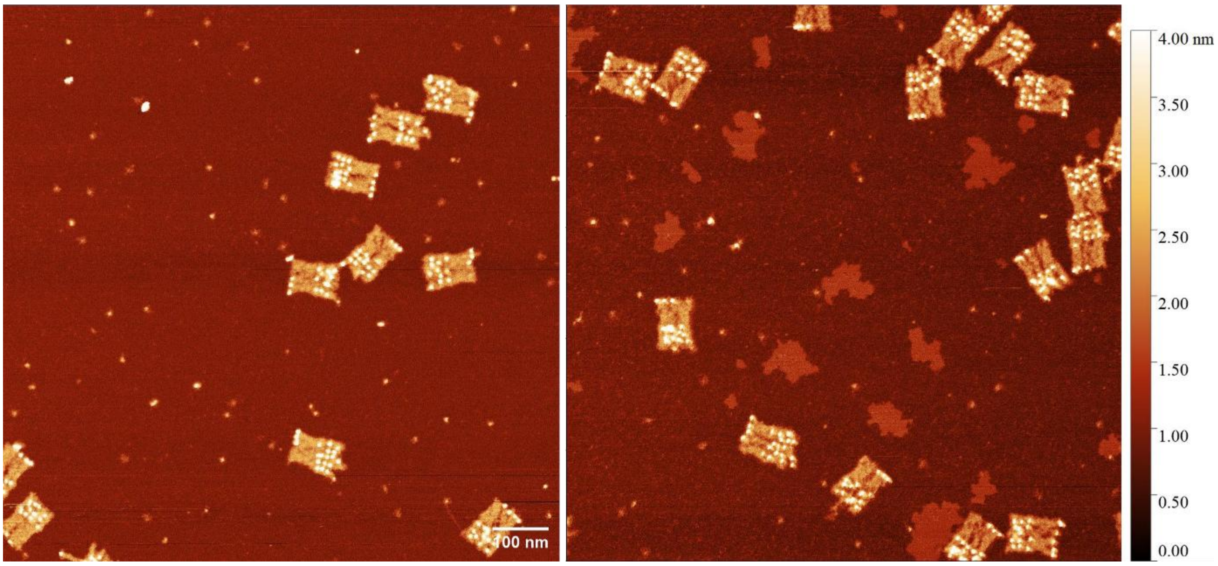

Figure S15: AFM images of DON rectangles with the 3 x 4 Bt array after stepwise SAv addition at a final concentration of 283 nM.

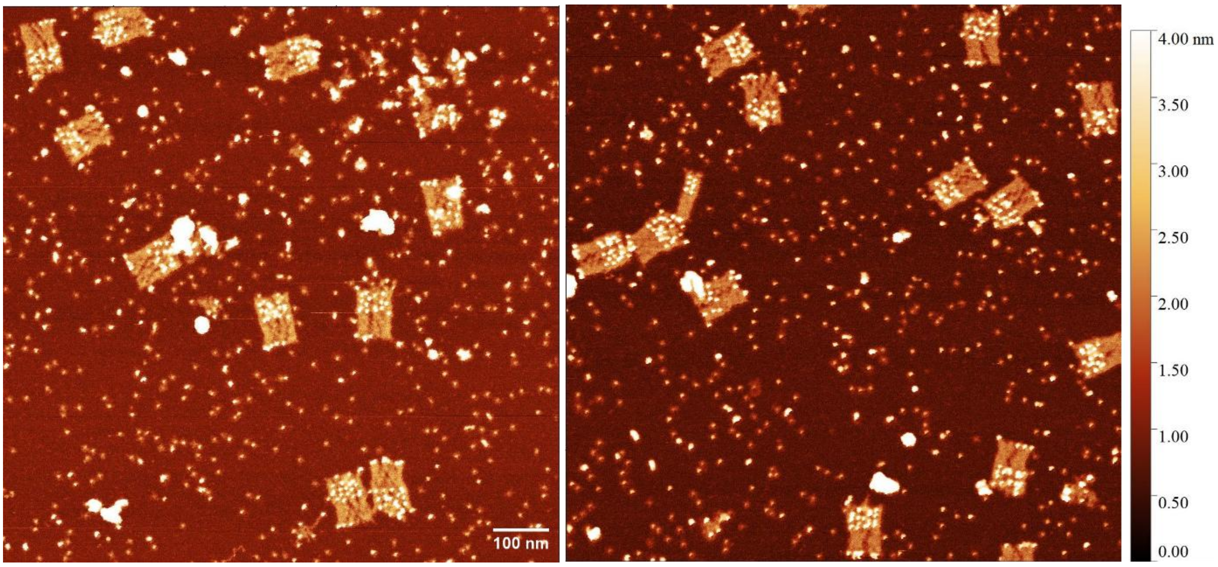

Figure S16: AFM images of DON rectangles with the 3 x 4 Bt array after stepwise SAv addition at a final concentration of 349 nM.

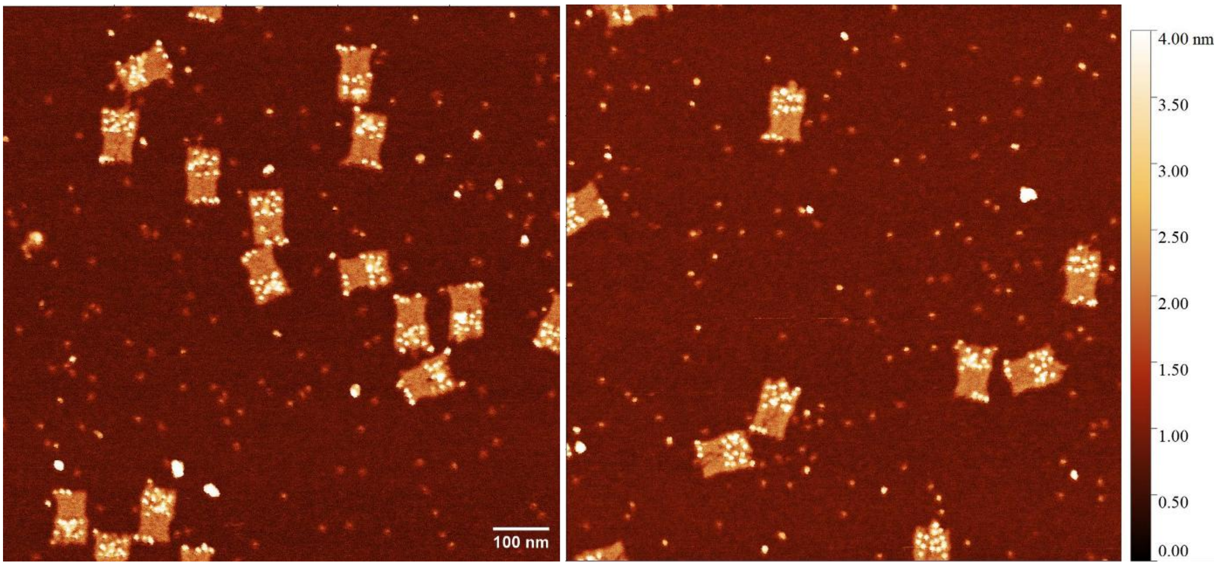

Figure S17: AFM images of DON rectangles with the 3 x 4 Bt array after stepwise SAv addition at a final concentration of 413 nM.
